# A suggested screening method for hypothyroidism in very preterm and/or very low birth weight neonates

**DOI:** 10.1590/1984-0462/2022/40/2020376IN

**Published:** 2022-05-06

**Authors:** Kayvan Mirnia, Sina Dindarian, Sedra Mohammadi, Parastoo Rostami, Hozan Mohammadi

**Affiliations:** a Tehran University of Medical Sciences, Tehran, Iran.; b Urmia University of Medical Sciences, Urmia, Iran.; c Tabriz University of Medical Sciences, Tabriz, Iran.

**Keywords:** Congenital hypothyroidism, Infant, premature, Thyrotropin, Thyroxine, Hipotireoidismo congênito, Recém-nascidos pré-termo, Tirotropina, Tiroxina

## Abstract

**Objective:**

To assess thyroid function in very preterm or very low birth weight (VLBW) neonates by measuring combination levels of thyroid-stimulating hormone TSH and free T4 (FT4)

**Methods:**

Inclusion criteria were defined as all very preterm (gestational age <32 weeks) or VLBW (birth weight ≤1500g) neonates with initial Thyroid Function Test (TFT) who were admitted to the Neonatal Intense Care Unit (NICU) of Taleghani Hospital, Tabriz, Iran, from March 2015 to March 2016. Exclusion criteria were the absence of initial TFT with any major congenital anomaly. The primary value of TSH was evaluated at 3–5 days, and mean levels of TSH with FT4 were measured at 2, 4, and 8-weeks.

**Results:**

Ninety-five neonates with a mean gestational age of 29.5 weeks were included, and the mean levels of thyrotropin and FT4 at postnatal week two were 4.4mIU/L and 1.4ng/dL, respectively. Two of the patients had serum TSH concentration >25mIU/L that was considered as permanent primary hypothyroidism. Among nine hypothyroxinemia cases, two had elevated TSH levels (10.8±0.4mIU/L at the end of 8 weeks) and normal FT4 concentration, and were considered transient hypothyroidism. Seven cases had normal TSH levels (1.6±1.0mIU/L at 2 weeks, 3.5±2.8mIU/L at 8 weeks) and low FT4 concentrations.

**Conclusions:**

Combined venous TSH and FT4 concentration at the end of the first postnatal month can be an efficient approach for detecting neonatal hypothyroidism.

## RESUMO

**Objetivo:**

Avaliar a função da tireoide em recém-nascidos muito prematuros ou de muito baixo peso por meio dos níveis de combinação de TSH e T4 livre (FT4).

**Métodos:**

Os critérios de inclusão foram: todos os recém-nascidos muito prematuros (idade gestacional <32 semanas) ou de muito baixo peso (peso ao nascer ≤500g) com teste de função tireoidiana inicial e que foram admitidos na Unidade de Terapia Intensiva Neonatal do Hospital de Taleghani, Tabriz, Irã, de março de 2015 a março de 2016. Os critérios de exclusão foram: ausência de TFT inicial com qualquer anomalia congênita importante.

**Resultados:**

95 neonatos com idade gestacional média de 29.5 semanas foram incluídos, e os níveis médios de tireotropina e FT4 na 2ª semana pós-natal foram 4.4mIU/L e 1.4ng/dL, respectivamente. Dois dos pacientes apresentavam concentração sérica de TSH >25mIU/L, considerada hipotireoidismo primário permanente. Entre nove casos de hipotiroxinemia, dois tinham níveis elevados de TSH (10.8±0.4mIU/L ao final de 8 semanas) e concentração normal de FT4 e foram considerados hipotireoidismo transitório. Sete casos tinham níveis normais de TSH (1,6±1,0mIU/L em 2 semanas, 3,5±2,8mIU/L em 8 semanas) e baixas concentrações de FT4.

**Conclusões:**

A concentração combinada de TSH e FT4 venoso no final do primeiro mês pós-natal pode ser uma abordagem eficiente para detectar hipotireoidismo neonatal.

## INTRODUCTION

Hypothyroidism in very preterm) gestational age <32 weeks) or very low birth weight (VLBW; birth weight ≤1500gr) neonates is more common than in term infants.^1^ Hence, adequate amounts of thyroid hormones (TH), such as Tri-iodothyronine (T3) and Thyroxine (T4), are not produced efficiently by the thyroid during the first weeks in neonates, which is more severe in low birth weight and small neonates.^2,3,4^ The fetal Hypothalamic-Pituitary-Thyroid Axis (HPTA) starts working by mid-gestation but continues to evolve until delivery. Therefore, during the first half of pregnancy, TH concentrations are low in the fetus, and the fetus is entirely dependent on maternal TH. The thyroid status of the mother and the placenta adjust the supply of TH to the fetus.^5^ Transient hypothyroxinemia is observed more often in infants born before gestation week 30 and is defined by a temporary postnatal reduction of TH with elevated levels of Thyroid-Stimulating Hormone (TSH).^6^ During the early stages of pregnancy, maternal hypothyroidism by itself and congenital hypothyroidism (CH) postpartum can both lead to significant neurodevelopmental disorders and cognitive impairment in neonates. The reason why hypothyroidism is more frequent in very preterm neonates is known to be related to several factors, including a sudden increase in the need for TH for thermogenesis, nonfunctional immature HPTA, thyroid with immature capacity, interruption of maternofetal transfer, non-thyroidal disease in very preterm infants, and iodine deficiency.^2,3,7^ It seems that the prevalence of the disease varies according to geographical and climatic characteristics.^8^ Three screening methods are used for CH, which are the following: I) Primary TSH method with screening card, II) Primary T4 method, and III) Combined primary venous TSH and T4 method. Neonate screening tests in Iran are based on the first method (Primary TSH method with screening card), which does not check free T4 (FT4) levels.^8,9^ In central hypothyroidism and hypothyroxinemia status with delayed elevation of TSH, primary TSH is not always elevated in very preterm and VLBW neonates. In some primary CH cases, TSH may be elevated at first and TSH may increase on follow-up tests (2, 4, and 8 weeks postnatal).^7,10^ Physicians’ clinical judgment is helpful and plays an important role in assessing CH. Therefore, in the case of normal or high thyroid test results, physicians must not ignore their clinical judgment and experience.^11^ Also, hypothyroidism can be acquired even after routine neonate screening.^7,9,10^ In this study, we focused on the efficiency of combined TSH and FT4 venous concentration after one month in detecting CH in very preterm and/or VLBW neonates that had normal or slightly elevated TSH levels during neonate screening.

## METHOD

All very preterm (gestational age <32 weeks) and/or VLBW (birth weight ≤1500gr) neonates with initial Thyroid Function Test (TFT) who were admitted to the Neonatal Intensive Care Unit (NICU) of Taleghani Hospital, Tabriz, Iran from March 2015 to March 2016 were included. Out of 222 patients, only 95 cases who had initial TFT were included in the study. Exclusion criteria were determined as neonates without initial TFT and with any major congenital anomalies. We obtained the clinical data of these 95 cases from medical records and evaluated their perinatal and postnatal factors, retrospectively, and tried to follow all of them in NICU) our follow-up clinic) for three years. The demographic factors and Apgar score at 1 and 5 minutes are included in Table 1.

**Table 1:** Demographic characteristics of the patients studied.

	Congenital hypothyroidism group (n=11)	Non congenital hypothyroidism group (n=84)	p-value
Birth weight (g)	1262±302	1367±267	0.423
Male sex [n(%)]	4 (36)	45 (53)	0.283
Maternal age (years)	24±6	25±6	0.910
Gestational age (weeks)	29±2	29 ±1	0.394
Apgar score at 1 minute	7.6±1.6	7.5±0.9	0.139
Apgar score at 5 minutes	8.5±1.6	8.7±1.4	0.950
Preeclampsia [n(%)]	7 (8)	2 (18)	0.294
Maternal gestational diabetes [n(%)]	0 (0)	1 (1)	0.716
Multiple gestation [n(%)]	1 (9)	29 (34)	0.880
Delivery by C-section [n(%)]	6 (54)	52 (63)	0.569

**Figure 1.**
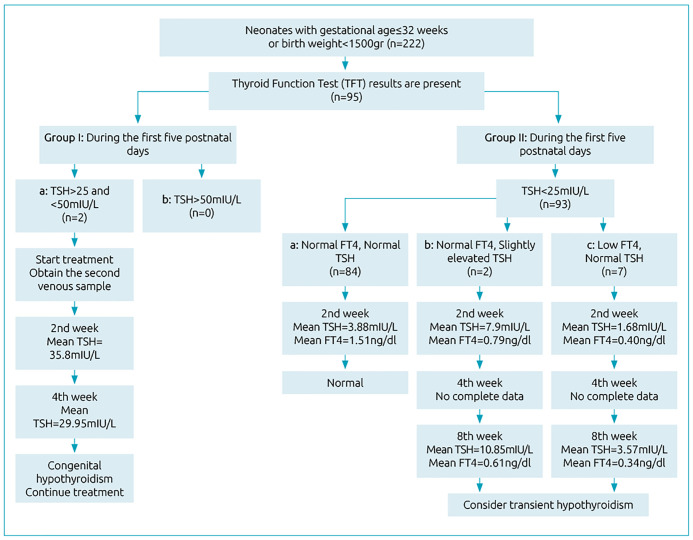
Study flowchart.

According to the Iranian Ministry of Health guidelines, this study was carried out in accordance with national guidelines (Iran High-Risk Infants Follow-up Service Package based on the article by Heidarzadeh et al.).^12^ In Iran, according to this guideline, we sample all very preterm and the VLBW neonates four times at 3–5 days, 2 weeks, 4 weeks, and 8 weeks postnatal only by measuring TSH. We requested that 4-weeks postnatal sampling (end of the first month) be replaced with FT4 test besides routine TSH checking. There will be only two sampling times if TSH was below 5mIU/L: the first at the 3–5 days postnatal by checking routine TSH only and the second at the 4weeks postnatal by adding FT4 to routine TSH checking.^12^ The TSH values of the neonates during the first five postnatal days were checked through venous sampling. Neonates with TSH levels higher than 50mIU/L were considered to have primary CH and treatment was initiated. If TSH was between 25 and 50mIU/L, we obtained another venous sample and started treatment. If the second sample confirmed the same result, the neonate was considered to have primary CH. In neonates with TSH levels lower than 25mIU/L and higher than 5mIU/L, we checked FT4 and TSH levels at 2 weeks, 4 weeks, and 8 weeks postnatal (Tables 2 and 3). Neonates with normal TSH and FT4 levels were considered normal, so there was no reason to follow them. In neonates with slightly elevated TSH and low FT4 levels, and in neonates with normal TSH and low FT4 levels, we double-checked their TSH and FT4 levels at 4 weeks and 8weeks postnatal. When TSH levels were normal and FT4 levels were low, we considered it transient hypothyroidism, which required treatment.^13,14^ (Figure 1). TSH and FT4 levels were checked by Eliza reader model stat fax 2100 made in the USA.

**Table 2:** Patients’ mean gestational age, thyrotropin levels, and free T4 levels based on weight class at 2 weeks postnatal.

Number of patients	Birth weight (g)	Gestational age (weeks)	Thyrotropin* (mIU/L)	FT4 * (ng/dL)
10	750–1009	27.0	3.2	1.1
23	1010–1269	29.0	4.3	1.3
40	1270–1529	30.0	5.7	1.5
17	1530–1789	30.2	3.0	1.3
4	1790–2049	31.0	2.9	1.5
1	2050–2309	30.0	2.1	1.9

FT4: free T4; TSH normal value: 0.5–5mIU/L; FT4 normal value: 0.6–2ng/dL; *mean values.

**Table 3:** Groups of neonates with abnormal TSH or FT4 levels during postnatal tests.

Patient No.	Group	Gestational age (weeks)	Birth Weight (g)	TSH level (mIU/L) 2nd week	FT4 level (ng/dL) 2nd week	TSH level (mIU/L) 4th week	FT4 level (ng/dL) 4th week	TSH level (mIU/L) 8th week	FT4 level (ng/dL) 8th week	Diagnosis in follow-up
1	Ia	31	1450	33.2	–	28.7	–	–	–	(+)
2	Ia	31	1270	38.4	–	31.2	–	–	–	(+)
3	IIb	30	1210	9.7	0.8	–	–	10.5	0.4	(+)
4	IIb	31	1450	6.1	0.7	–	–	11.2	0.7	(+)
5	IIc	31	1150	3.0	0.7	–	–	5.8	0.4	(+)
6	IIc	26	750	2.5	0.0	12.2	0.4	8.2	0.4	(+)
7	IIc	29	1500	1.0	0.6	3.1	0.6	2.9	0.4	(+)
8	IIc	27	950	0.5	0.5	–	–	1.0	0.3	(+)
9	IIc	31	1560	0.2	0.1	–	–	0.0	0.1	(+)
10	IIc	28	900	2.3	0.2	3.1	0.4	4.4	0.3	(+)
11	IIc	31	1700	2.2	0.6	–	–	2.7	0.3	(+)

Group Ia: TSH>25mIU/L (during the first five postnatal days): Group Ib: TSH>5mIU/L (during the first five postnatal days); Group IIa: 0.5<TSH<5mIU/L, Free T4>0.6ng/dL (at week 8 postnatal); Group IIb: TSH>5mIU/L, Free T4>0.6ng/dL (at week 8 postnatal); Group IIc: 0.5<TSH<5mIU/L, Free T4<0.6ng/dL (at week 8 postnatal); TSH: thyroid-stimulating hormone; FT4: free T4.

The morbidity and outcome variables were: respiratory distress syndrome (RDS), seizure, intraventricular hemorrhage (IVH) (≥grade III),^15^ necrotizing enterocolitis (NEC) (modified bell stage≥II),^16^ sepsis (positive culture in addition to the presence of clinical signs of systemic infection),^17^ bronchopulmonary dysplasia (BPD) (oxygen dependency during the first 28 days of life),^18^ retinopathy of prematurity (ROP) (high stage requiring laser therapy),^19^ and Patent Ductus Arteriosus (PDA) (Table 4).

**Table 4:** Morbidity and outcomes.

	Congenital Hypothyroidism group (n=11)	Non congenital hypothyroidism group (n=84)	p-value
RDS [n(%)]	6 (54)	60 (71)	0.253
PDA [n(%)]	4 (36)	14 (16)	0.117
Seizure [n(%)]	1 (9)	0	0.005
Sepsis [n(%)]	2 (18)	18 (21)	0.804
IVH (≥grade III) [n(%)]	1 (9)	16 (19)	0.418
NEC (≥stage II) [n(%)]	0 (0)	2 (2)	0.605
BPD (≥moderate) [n(%)]	4 (5)	0 (0)	0.460
ROP (requiring laser surgery) [n(%)]	0 (0)	2 (2)	0.605

RDS: respiratory distress syndrome; PDA: patent ductus arteriosus; IVH: intraventricular hemorrhage; NEC: necrotizing enterocolitis; BPD: bronchopulmonary dysplasia; ROP: retinopathy of prematurity.

We performed a Student’s *t*-test for continuous variables and a chi-square test for nominal variables. Statistical analysis was performed using IBM *Statistical Package for the Social Sciences* (SPSS) 22. Data are given as mean±standard deviation (SD). p<0.05 were considered statistically significant.

The present study was registered with IR.TBZMED. REC.1397.589 in the Ethics Committee of Tabriz University of Medical Sciences.

## RESULTS

**Figure 2.**
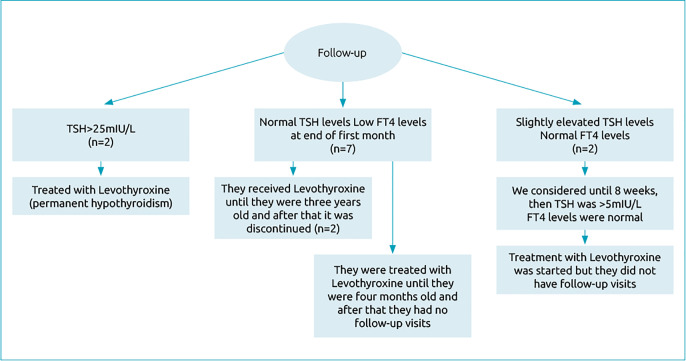
Follow-up flowchart.

Ninety-five neonates with a mean gestational age of 29.5 weeks were included and divided into two groups based on TSH levels during the first five postnatal days. At the 2^nd^ postnatal week, the mean levels of thyrotropin and FT4 based on the weight were 4.4mIU/L and 1.4ng/dL, respectively (Table 2). Two of the patients who had TSH>25mIU/L were considered a high suspicious group. Their 2-week and 4-week follow-up were done only by measuring the level of TSH (35.8±3.6mIU/L), and they were diagnosed as having permanent primary hypothyroidism. These two cases were twins, and their follow-up showed they were under treatment with Levothyroxine (15μgr/kg/day PO). Out of 97.8% (n=93) of the patients with TSH<25mIU/L, 88.4% (n=84) had normal initial TFT. Among nine cases of hypothyroxinemia, two had elevated TSH levels (10.8±0.4mIU/L at the end of 8 weeks) and normal FT4 concentration; they were treated with Levothyroxine (15μgr/kg/day PO). After 8 weeks of follow-up, they were considered to have transient primary hypothyroidism. Eventually, seven cases had normal TSH levels (1.6±1.0mIU/L at 2 weeks, 3.5±2.8mIU/L at 8 weeks), and low FT4 concentrations (Figure 1). They were also followed for 8 weeks to monitor transient hypothyroidism. Two of them were followed until the end of the follow-up period and received Levothyroxine (15μgr/kg/day PO) until the age of three years. After that timespan, the drug was discontinued due to their normal ASQ, but their height was in the 5^th^ percentile approximately. The other five cases were followed and received Levothyroxine (15μgr/kg/day PO) until the age of four months, but unfortunately, after this timespan, they did not come back to our NICU follow-up clinic (Table 3 and Figure 2). Our cut-off point for hyperthyroidism was TSH below 0.5mIU/L. And among the patients being followed, we reduced the dose in two cases. Also, we observed no clinical symptoms of hyperthyroidism among our patients.

## DISCUSSION

Based on the results of this study, checking both TSH and FT4 at the end of the first month (4 weeks postnatal) in preterm neonates helps to provide an accurate diagnosis of hypothyroidism and prevents misdiagnosis. Taleghani Hospital is a referral maternity hospital that admits patients from four provinces in the west and north-west of Iran. As shown in the flowchart, 127 cases were not able to return for a visit to our follow-up clinic due to long distances. We treated neonates with high TSH and normal FT4 levels after the first month of postnatal age. It can be seen in Figure 1 that checking FT4 levels in the first days may not be helpful to diagnose hypothyroidism, but checking it at the end of the first month or 8 weeks postnatal can guide physicians to diagnose transient hypothyroidism. For example, two cases in our study were followed and treated with Levothyroxine (15μgr/kg/day PO) for three years. Levothyroxine (15μgr/kg/day PO) was maintained until their TSH test result came back normal. The remaining patients were followed and received the drug for about four months. During follow-up, we monitored the neonates for hyperthyroidism symptoms, but no significant symptoms were detected. We also followed neonates with a slight elevation in TSH levels and normal FT4 levels at the end of the 8-week period postnatal. Among the patients with a slight elevation in TSH levels and normal FT4 levels at the end of 8 weeks, only two cases were recognized as hypothyroidism and were treated with Levothyroxine (15μgr/kg/day PO). This suggests that in these cases, with a slight elevation in TSH levels (TSH >5 and <10mIU/L) and normal FT4 levels, the correct approach would be to wait and follow the patient for 8weeks. This approach is important because any increase in TSH levels after 8 weeks will lead the physician to a diagnosis of hypothyroidism and will prevent the condition from being missed. The greatest challenge in our study was answering the following question: Do we need to check FT4 levels at the end of the first month in very preterm neonates? Iranian Minister of Health guidelines do not advocate this treatment, but most of our colleagues in various NICU centers check FT4 levels in practice. Another challenge was whether we could rely on tests using screening cards in NICU for very preterm neonates. We concluded that we cannot rely on screening cards alone, and we have to use venous samples as well and follow some cases for 8 weeks to make proper decisions. Although TSH levels surge and reach a baseline (less than 5mIU/L) 24 to 48 hours after birth, this decline may not occur in very premature neonates, and TSH levels may remain high at 6 to 10mIU/L. In such cases, we do not start treatment immediately, and we suggest following the neonate every week until TSH levels are lower than 5mIU/L within 4 to 8 weeks. If levels did not decrease and remained slightly higher than normal or even increased at the end of the 8-week period, we started treatment. This study was also not able to suggest any specific approach to manage patients with TSH levels between 10 and 25mIU/L.^20,21^

There are various methods for screening CH in different countries. These screening methods comprise TSH, FT4, or combined TSH and FT4 methods. For instance, combined TSH and FT4 methods are used in some states of the United States in which TSH and FT4 levels are checked in VLBW neonates at 2, 6, and 10 weeks postnatal, as well as in neonate screening. This procedure is set by the New England Neonate Screening Program’s policies and has been followed since 1996. Since then, CH screening has been implemented in Iran, and mainly the primary TSH approach has been used. In the current study, we measured TSH levels during the first five postnatal days, but we also checked FT4 levels in neonates with TSH<25mIU/L in 2, 4, and 8 weeks postnatal to detect very preterm or VLBW neonates with late-onset transient hypothyroidism. Dussault et al. aimed to compare the effectiveness of a primary TSH approach with a primary FT4 approach in a Quebec study.^22^ Using a primary TSH approach, two cases of permanent CH among 93,000 neonates were missed, while the primary FT4 approach was able to detect these two cases. The primary FT4 approach also resulted in a case of permanent CH being missed. However, the primary TSH approach was able to detect this case. The American Academy of Pediatrics^23^ stated that the primary TSH approach missed delayed TSH elevation in neonates with Thyroid-Binding Globulin deficiency, central hypothyroidism, and hypothyroxinemia. The limitations of the primary FT4 approach also include missing CH in neonates with normal FT4 concentrations and delayed increased TSH levels. As the delayed elevation of TSH levels is common in neonates with low or very low birth weight (VLBW), physicians should not neglect the limitations of both approaches. Mandel et al.^24^ also agree with this statement by suggesting that in addition to the primary TSH screening method, FT4 levels should also be measured with a second blood sample in all neonates with low or VLBW. Our study detected nine cases of hypothyroidism that had normal or slightly elevated TSH levels during five postnatal days and 2 weeks postnatal. We could detect these cases using FT4 screening in addition to a primary TSH approach. In the study conducted by Mitchell et al.,^25^ nine cases of late-onset transient hypothyroidism were reported. Also, Frank et al.^26^ detected that two out of 48 cases with VLBW had late-onset hypothyroidism. These reports show the importance of considering more efficient screening CH methods rather than applying a primary TSH or FT4 approach only. Based on the Iranian Ministry of Health guidelines, neonates are sampled four times, but neonates are sampled twice only in the protocol suggested by us. As a result of this protocol, we will not inflict more sampling on neonates and, at the same time, we increase the accuracy of diagnostic tests by adding FT4 levels to routine TSH checking at the 4-week postnatal sampling. In our study, we assessed the correlation between seizure and thyroid dysfunction as well. We observed a statistically significant correlation, but further studies are recommended because of the low number of cases. This study shows we need to design a new protocol for managing different levels of TSH and FT4, comparing screening card TSH to venous TSH in very premature neonates, and following them in the long term as to neurodevelopmental aspects. We believe that the screening card method is not a reliable method for screening premature neonates.

This study also had some limitations, which include: our healthcare center is a specialized hospital for referral patients, and many neonates were admitted from different provinces. Consequently, it was not possible to follow all neonates due to long distances. In some cases, they were discharged before the second week of life and they did not return to our clinic, being withdrawn from the study. Meanwhile, our study revealed at least two cases that required treatment for three years. We believe treating even one case is cost-beneficial. Besides, the external validity of the study is not clear because the results of 127 neonates were lost.

In a study by Lee et al.,^7^ no significant relationship was observed between thyroid dysfunction and demographic factors, including gestational age, birth weight, sex, Apgar score at 1 and 5 minutes, maternal Pregnancy-Induced Hypertension (PIH), and GDM, while they observed a significant correlation between the state of being Small for Gestational Age (SGA) and thyroid dysfunction. We did not assess the relationship between thyroid dysfunction and the state of being SGA in our study, but other findings were similar to our results. We also checked some other demographic factors which included maternal age, type of delivery, and twins to observe their potential correlation with thyroid dysfunction. The correlation between these variables was insignificant as well. Some studies are assessing the correlation between RDS and thyroid dysfunction. In the studies conducted by Cuestas et al.^27^ and Abuassi et al.,^28^ a correlation between RDS and thyroid dysfunction was detected. On the other hand, Williams et al.^29^ and Lee et al.^7^ stated no correlation between these two factors. Our study findings also suggested that there is no statistically significant correlation between RDS and thyroid dysfunction. Thus, the results of the present study are controversial, and there seems to be a need for further investigation in this field. In the current study, we found that the correlation between thyroid dysfunction and some morbidities such as sepsis, IVH, NEC, PDA, BPD, and ROP is not significant. Lee et al.^7^ also agree with these findings. However, Abuassi et al.^28^ stated that thyroid dysfunction could be induced by late-onset sepsis, IVH, and NEC, and the main reason for this phenomenon may be acute inflammatory cytokine response. Carrascosa et al.^1^ also suggested that neonates with PDA are more susceptible to thyroid dysfunction.

We recommend using TSH venous samples in very preterm and VLBW neonates after 72 hours of hospital admittance. If TSH levels are between 6 and 10mIU/L, then neonates should be checked every week for 4 weeks. Then TSH and FT4 levels are checked at the end of the first month in all very preterm and VLBW neonates. If TSH levels are still high or elevating after 8 weeks while FT4 levels are normal, we suggest starting treatment (Levothyroxine (15μgr/kg/day PO)). If FT4 levels decreased after 4 weeks while TSH levels are normal, we recommend starting treatment (Levothyroxine (15μgr/kg/day PO)). Measuring FT4 levels during the first days of hospital admittance may not help in the diagnosis of hypothyroidism. As a result of this protocol, we will not inflict more sampling on neonates. At the same time, we increased the accuracy of diagnostic tests by adding FT4 to routine TSH checking at the 4week postnatal sampling (the end of the first month), and the combined TSH and FT4 assessment method at 4 weeks postnatal can be an efficient and cost-effective approach for detecting hypothyroidism in neonates and preventing the lack of a diagnosis and its adverse side effects.
